# Multiple drug resistance of *Listeria monocytogenes* isolated from aborted women by using serological and molecular techniques in Diwaniyah city/Iraq

**DOI:** 10.18502/ijm.v12i4.3933

**Published:** 2020-08

**Authors:** Firas Srhan Abd Al-Mayahi, Saja Mahdey Jaber

**Affiliations:** 1 Department of Biology, College of Science, University of Al-Qadisiyah, Diwaniyah, Iraq; 2 Department of Science, School of Nursing, University of Al-Qadisiyah, Diwaniyah, Iraq

**Keywords:** *Listeria monocytogenes*, Enzyme-linked immunosorbent assay, Polymerase chain reaction, Antibiotic

## Abstract

**Background and Objectives:**

The study was sought to detect the effect of *Listeria monocytogenes* on pregnant Iraqi women at Al-Diwaniya hospitals and determination of virulence genes and antimicrobial susceptibility of isolates.

**Materials and Methods:**

360 specimens including blood, urine, vaginal and endocervical were collected from 90 patients with spontaneous abortions. Blood samples were displayed to immunological study and remaining specimens were subjected to bacteriological diagnosis. PCR was used to determine the virulence factors and antimicrobial resistance genes.

**Results:**

Fifteen positive samples (16.6%) of patients and thirteen isolates (14.5%) from patients were recognized based on ELISA and PCR assay respectively. The general isolation of *L. monocytogenes* strains in cases of abortive women was 13/270 (4.8%). *L. monocytogenes* strains were highly virulent because of presence of virulence factors associated genes, namely *actA, hlyA, plcA* and *prfA* in all strains. Multiple drug resistance (MAR) index values of 15.4% of isolates were >0.2.

**Conclusion:**

It is necessary for conducting susceptibility testing and to select the suitable antibiotics and avoid the effects of these bacteria in pregnant women.

## INTRODUCTION

*Listeria monocytogenes* is a facultative anaerobe, which capable of surviving in the presence or absence of oxygen, the bacteria is a Gram-positive and rod-shaped bacterium (1). It can be isolated from foods of animal origin, decaying vegetables, and from the faeces of mammals, birds, and other animals (2). *Listeria monocytogenes* affect pregnant women and causes abortion (3). The main route of infection is consuming food contaminated with *L. monocytogenes*, and eventually adverse impact on human health, with increases in rates of fatality (4). In addition, unpasteurized milk and unwashed fresh vegetables is another way to transmit the infection to human. However, the infection transfer from mother to child can also occur in utero or at birth (5). These bacteria are more commonly occur in ageing adults, immune-compromised people, HIV affected individuals, and pregnant women (6).

Listeriosis can be diagnosed through enzyme-linked immunosorbent assay (ELISA) as IgG antibodies raises against listeriolysin which may be a contribution in both invasive and non-invasive infections (7, 8). Moreover, various virulence genes are significant in the pathogenesis of these bacteria, such as phosphatidylinositol phospholipase C *(plcA)* invasive associated protein *(iap)* hemolysin *(hlyA)*, and actin polymerization protein *(actA)* (9). Researchers have investigated these genes in this bacteria involved in abortions (10–12).

There is little information on listeriosis in aborted Iraqi women. The problem of abortion or miscarriage in Iraq has been increasing because there is no systematic national surveillance database to record the microbial aetiology of abortion and antibiotic resistance profile to quantify the problem. Therefore, this work aimed (i) to identify *L. monocytogenes* in abortive women in Diwaniyah city/Iraq who reacted in ELISA: (ii) to isolate and identify *L. monocytogenes* and subsequently genotype the isolates by PCR targeting the virulence factors genes, and (iii) to assess the severity of the effect of listeriosis in Iraqi abortive women and antibiotic susceptibility (AS) pattern.

## MATERIALS AND METHODS

### Experimental design.

**Fig. 1.**
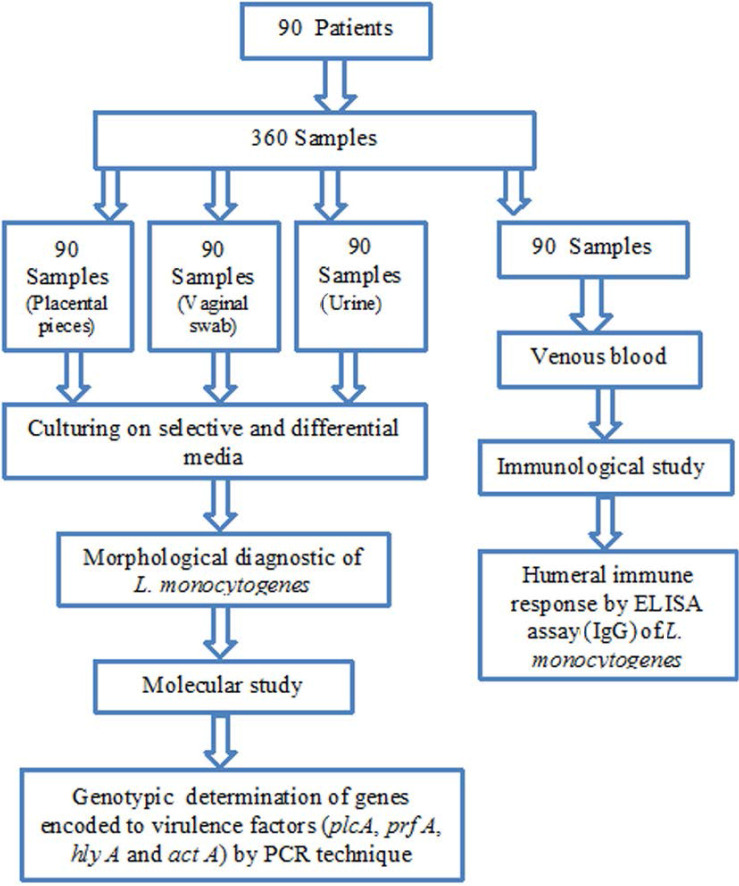
Flow chart of research design

The total experimental design carried out in this research and presented in Fig. 1. The methods were performed based on the samples collected from patients including blood, urine, vaginal swab and endocervical, and theses samples yielded bacteria that identified as *L. monocytogenes* which are underwent to immunological and molecular study.

### Specimens.

The samples (n= 360 specimens) were collected from 90 patients with spontaneous abortions from the period of January to May 2019. The samples were comprised of equal blood, urine, vaginal swab and endocervical swab in Maternity and Children teaching hospital at Al-Diwaniya city. Two millilitres of each blood sample was put in a sterilized tube and left at room temperature for 30 min in order to from a clot. Then, the tubes were centrifuged at 6000 RPM for 10 minutes, after that, the partition of the serum was poured into a spotless tube then stored at −20°C until used for ELISA test. At the same time, a vaginal, urine and endocervical swabs were taken from all bleeding woman in this study. Endocervical swabs were acquired by inserting the swab into the endocervical canal until greatest of the tip is no longer visible, and inflexibly rotate the swab for (15–20) seconds and put it in a sterilized test tube as well as pieces of the placenta was taken and also put it in a sterilized test tube. The swabs and placenta were sent directly to the laboratory for bacterial culture. The identification of bacteria was conducted through phenotypic and genotypic characters (13). Lastly, the patients’ information was recorded such as age, region live, gestational age and abortion recurrence.

### Immunological diagnosis.

The immunological procedure was performed according to the manufacturer’s instructions (*L. monocytogenes* IgG-ELISA kit) (Diatheva, Italy). In brief, the principle of the assay is microtiter strips loaded with listeriolysin O (LLO) are incubated with composed samples. Throughout this incubation step, anti-listeriolysin O antibodies bind to the antigen production-specific complexes in the end (14).

### Phemotypic identification of *L. monocytogenes.*

All bacterial isolates were identified using conventional cultural, phenotypic characters (Gram staining, catalase test, motility test at 25°C and 37°C, growth at 4°C, nitrate reduction, MR/VP test, and ß-hemolytic activity (15).

### DNA extraction.

Salting out method was used to extract deoxyribonucleic acid from *L. monocytogenes* cells according to Pospiech and Neumann (16).

### PCR assay.

The reaction was performed through a 25 μl mixture including 5 μl of deoxyribonucleic acid, 12.5 μl Go Tag Green Master mix (Promega, USA), 2.5 μl of each primers (Macrogen, Korea) and PCR water 2.5 μl. The amplification was performed in a thermocycling system (Biometra, Germany). Primer sequence and the amplification conditions of each (*plcA, hlyA, actA* and *prfA*) primers virulence genes are listed in Table 1. The amplifications were electrophoresed (Biometra, Germany) through a 1.5% agarose gel pre-exposed to ethidium bromide. Then products of PCR were visualized under a UV imager (Biometra, Germany). Finally, two molecular weight DNA markers were used (Ladder 100 bp Promega, and Ladder 10000 bp Kappa Biosystem, USA).

**Table 1.** Oligonucleotides sequence used of virulence factors gene targets in this study.

**Gene target**	**Forward primer (5 to 3)**	**Reverse primer (5 to 3)**	**Amplicon size (bp)**	**Reference**
*plcA*	CTGCTTGAGCGTTCATGTCTCATCCCCC	CATGGGTTTCACTCTCCTTCTAC	1484	17
*prfA*	AATCGTACAGGACGATGAACCC	GGTATCACAAAGCTCACGAG	571	18
*hlyA*	GCAGTTGCAAGCGCTTGGAGTGAA	GCAACGTATCCTCCAGAGTGATCG	456	19
*actA*	CGCCGCGGAAATTAAAAAAAGA	ACGAAGGAACCGGGCTGCTAG	839	20

### Antimicrobials sensitivity test.

Antibiotic susceptibility testing of *L. monocytogenes* isolates was performed using standard modified disc diffusion method (Kirby-Bauer) (21) on Mueller-Hinton agar (MHA) (Oxoid, UK) with 20 mg/L β-NAD and 5.0% of horse blood. The selection of antibiotic discs and determination of susceptibility were performed according to EUCAST (22). The susceptibility of ampicillin (AM, 2 μg), penicillin (P, 1IU), erythromycin (E, 15 μg), meropenem (MEM, 10 μg) and cotrimoxazole (SXT, 1.25–23.75 μg) were tested to *L. monocytogenes* strains. Multiple antibiotic resistance (MAR) index was detected for each strain by dividing the number of drugs against which the strain displayed resistance above the total drugs tested (23).

### Analysis.

The χ^2^ (Chi-square) tests were applied to determine the statistical significance of the data. P value of < 0.01 or < 0.05 was considered significant, Prism 5 (Graph Pad Software Inc., San Deigo, CA, USA).

## RESULTS

The present study was carried out to detect and identify *L. monocytogenes* isolated from abortive women. During January to May 2019, 15 (16.6%) and 13 patients (14.5%) were found positive for listeriosis based on the results of ELISA and PCR assay respectively. The cases were in the groups of 21–30 years (n=7, 53.8%), 1^st^ trimester (n=9, 69.2%), rural (n=11, 84.6%) and recurrent (n=7, 53.8%). The statistical analysis revealed a significant different in abortive woman frequency among the studied age group, 1^st^ trimester and recurrence in ELISA and PCR assay (P< 0.05), while there was a significant difference between living area of patients and results of PCR (P< 0.05) (Table 2).

**Table 2.** Characteristics of patients infected with *L. monocytogenes* phenotypically and genotypically

**Patients profile**	**Status**	**No. of patients (%)**	**ELISA No. of positive (%)**	**PCR No. of positive (%)**
Age group (Years)	≤ 20	20 (22.2)	1 (6.7)	2 (15.4)
	21–30	46 (51.1)	9 (60)	7 (53.8)
	31–40	21 (23.4)	4 (26.6)	3 (23.1)
	≥ 41	3 (3.3)	1 (6.7)	1 (7.7)
Gestational age	1^st^ Trimester	56 (62.2)	11 (73.3)	9 (69.2)
	2^nd^ Trimester	25 (27.8)	3 (20)	2 (15.4)
	3^rd^ Trimester	9 (10)	1 (6.7)	2 (15.4)
Living area	Rural	59 (65.6)	8 (53.3)	11 (84.6)
	Urban	31 (34.4)	7 (46.7)	2 (15.4)
Abortion recurrence	Single	51 (56.7)	5 (33.3)	6 (46.2)
	Recurrent	39 (43.3)	10 (66.7)	7 (53.8)
Total	Total	90 (100)	15 (16.6)	13 (14.5)

The rate of isolation for *L. monocytogenes* in cases with abortions was 13/270 (4.8%). Isolates of *L. monocytogenes* were screened for the presence of virulence factors associated with genes, namely *actA, hlyA, plcA* and *prfA* using conventional uniplex-PCR and universal specific primers, which their expected size fragment were 456, 571, 839 and 1484 bp respectively. The results of the PCR with consensus primers for the isolates of *L. monocytogenes* showed that all isolates were positive for the all tested genes (Table 3). According to source of the isolation, *L. monocytogenes* divided into three types: placental pieces 9 (69.2%), vaginal swab 3 (23.1%) and urine 1 (7.7%) (Tables 3 and 5 Significant difference was observed among antibiotics resistance and abortive woman frequency (P< 0.05). Antimicrobial susceptibility pattern of *L. monocytogenes* isolates was as follow: all strains were sensitive to meropenem, while 12 (92.3%), 11 (84.6%), 11 (84.6%) and 9 (69.2) isolates were sensitive to cotrimoxazole ampicillin, erythromycin, and penicillin, respectively (Table 3). The majority of resistance profiles were observed among isolates from placental specimens (n=5, 83.4%). There is a significant difference in abortive woman frequency depending on source of sample (P < 0.05). Moreover, statistical analysis found significant difference in abortive woman frequency among drugs resistance patterns depending on type of sample (P < 0.05).

**Table 3.** Morphological and biochemical tests, PCR and antibiotics patterns of *L. monocytogenes* strains in abortive women.

		**Morphological and biochemical tests**							**PCR patterns of virulence genes**				**Antibiogram resistant**
	
**No. of isolates**	**Source**	**Gram Staining**	**Catalase**	**Growth at 4°C**	**Hemolysis**	**Nitrate reduction**	**MR/VP test**	**Motility 25/37°C**	***plcA***	***prfA***	***actA***	***hlyA***	
LM1	PP	+	+	+	β	−	+/+	+/−	+	+	+	+	E, AM
LM2	PP	+	+	+	β	−	+/+	+/−	+	+	+	+	P
LM3	VS	+	+	+	β	−	+/+	+/−	+	+	+	+	P
LM4	PP	+	+	+	β	−	+/+	+/−	+	+	+	+	(−)
LM5	U	+	+	+	β	−	+/+	+/−	+	+	+	+	(−)
LM6	PP	+	+	+	β	−	+/+	+/−	+	+	+	+	P, AM, E
LM7	VS	+	+	+	β	−	+/+	+/−	+	+	+	+	(−)
LM8	PP	+	+	+	β	−	+/+	+/−	+	+	+	+	SXT
LM9	VS	+	+	+	β	−	+/+	+/−	+	+	+	+	(−)
LM10	PP	+	+	+	β	−	+/+	+/−	+	+	+	+	(−)
LM11	PP	+	+	+	β	−	+/+	+/−	+	+	+	+	P
LM12	PP	+	+	+	β	−	+/+	+/−	+	+	+	+	(−)
LM13	PP	+	+	+	β	−	+/+	+	+	+	+	+	(−)

P: placental pieces, V: vaginal swab, U: urine, P: penicillin AM: ampicillin, E: erythromycin, SXT: cotrimoxazole, (−): sensitive to all.

The results confirmed that 7 (53.8%) out of the 13 isolates of *L. monocytogenes* were susceptible to all drug. The remainders (n=6, 46.2%) were resistant from one to three of tested antibiotics (Table 4). Resistance to penicillin was detected in four isolates (30.7%), increase susceptibility to ampicillin and erythromycin were observed in two isolates (15.4%) for each one, cotrimoxazole resistance had observed in 1 (7.7%) isolates (Table 4).

**Table 4.** Dissamination patterns of drug resistance possessed *L. monocytogenes* strains.

**Drug**	**Susceptibility patterns No. (%)**		**Patterns of antibiogram resistant**		**No. (%)**
	
	**Resistance (R)**	**Sensitive (S)**	**R**	**S**	
Penicillin (P)	4 (30.8)	9 (69.2)	P	AM, MEM, E, SXT	3 (23.1)
Ampicillin (AM)	2 (15.4)	11 (84.6)	P,AM, E	MEM, SXT	1 (7.7)
Meropenem (MEM)	0 (0)	13 (100)	-	AM, MEM, E, SXT, P	7 (53.8)
Erythromycin (E)	2 (15.4)	11 (84.6)	E,AM	MEM, P, SXT	1 (7.7)
Trimethoprim/	1 (7.7)	12 (92.3)	SXT	AM, MEM, E, P	1 (7.7)
Sulfamethoxazole (SXT)					

**Fig. 2.**
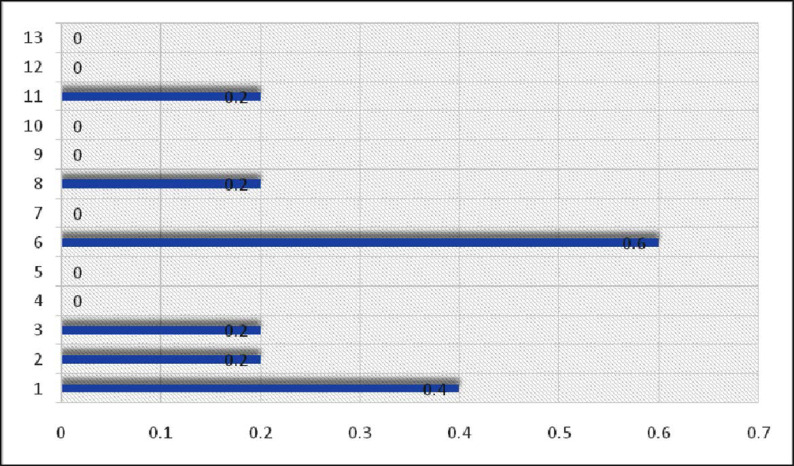
MAR indexes of *L. monocytogenes* strains in Iraqi aborted women

Multiple antibiotics resistance (MAR) index is a tool that reveals the spread of bacterial resistance in a given population. In this paper, the antibiogram was used to calculate the MAR indices. The frequency of MAR index is shown in Fig. 2.

## DISCUSSION

*Listeria monocytogenes* is usually found in dust, soils, water, sewage and decaying vegetation including animal feeds and silage, from where it enters the food chain. It can cause encephalitis, septicemia and abortion with mortality rates ranging between 80% and 99% primarily in neonates, immuno-compromised adults and pregnant women (24). PCR technique is a rapid and sensitive method for identification of *L. monocytogenes* and detection of its virulence factor gene *(hly)*. Diagnosis of Listeriosis can be achieved using ELISA and molecular techniques (25). Serological analysis led to the identification of *L. monocytogenes* in 15 (16.6%) of patients, while in 13 (14.5%) of patients by bacteriology (Table 2).

In Iraq, the presence of *L. monocytogenes* has been documented in women with spontaneous abortion by Yousif and Al-Shamari (26) who identified 13 isolates of *L. monocytogenes*, 6 of them from placental pieces and vaginal swabs.

In our study, the majority of *L. monocytogenes* isolates were detected from patients in the age group 21–30 years 7 (53.8%), 1^st^ trimester 9 (69.2%), rural 11 (84.6%) and 7 (53.8%) abortion recurrence of women (Table 2).

The miscarriage of an early pregnancy is the commonest medical complication in humans, with one in two conceptions lost before the end of the first trimester. Most conceptions are lost during the first month after the last menstrual period and are often undetected. Our results were expected according to the fact that the rate of clinical pregnancy loss is known to decrease with gestational age from 25% at 5–6 weeks to 2% after 14 weeks (27). The most common cause of spontaneous abortion during the first trimester is chromosomal abnormalities of the embryo or fetus, diabetes normal problem and infection (28).

Phenotypic identification tests are still misleading and insufficient compare with PCR (11). The determination of a single gene associated with virulence using PCR technique always is not adequate to its identity and access to the truth of its pathologic and genetic content, *L. monocytogenes* have different gene patterns in the majority of its strains (9). In this work, all 13 *L. monocytogenes* strains contained *actA, hlyA, plcA* and *prfA* genes encoding virulence factors. Also, Heidarzadeh et al. (12) documented the existence of the *actA* and *hlyA* genes in all *L. monocytogenes* isolates with spontaneous abortions. Whereas, Kaur et al. (11) found that 2 (50%) *L. monocytogenes* isolates carried all genes of this research plus *iap* gene. Rezaei et al. (29) documented the existence of the *hlyA* gene in all *L. monocytogenes* isolates with spontaneous abortions. On the other hand, the presence of *hlyA* gene was detected in only 2 (40%) out of five *L. monocytogenes* isolates while 3 lacked this gene (30) probably due to mutation (31). Therefore, determination of these pathogenic potentials is the main objective in *L. monocytogenes* infections (9). Listeriosis may be accompanied with either an increase in virulence due to the presence of many of responsible genes, this is in line with results of this study, or decreased in virulence due to spontaneous mutations in 1 or 2 virulence genes (32). The current global crisis of antimicrobial resistance (AMR) among important human bacterial pathogens has been amplified by an increased resistance prevalence. In recent years, the increasing resistance of *L. monocytogenes* strains to antibiotics has become a serious problem, especially with those used during standard therapy of patients with diagnosed listeriosis (33).

Seven isolates in this study (53.8%) were susceptible to tested antibiotics but 6 (46.2%) were resistant to at least one antimicrobial tested. This is different from the results reported by Lotfollahi et al. (10). In their study only 2 (22.2%) *L. monocytogenes* isolates were susceptible to tested antibiotics, and 7 isolates from abortive women (77.8%) were resistant to at least one drug. In this investigation the distribution of resistance among the isolated *L. monocytogenes* showed the frequency of resistance was 30.8% (n = 4) to penicillin, with resistance 15.4% (n = 2) to each ampicillin, erythromycin and 7.7% (n = 1) were resistance to cotrimoxazole. In contrast, Lotfollahi et al. (10) found all isolates (100%) were non-susceptible to penicillin, including 77.8% resistant and 22.2% intermediate resistant, whereas all isolates (100%) were sensitive to ampicillin, erythromycin and trimethopirim. In another study from Italy, all isolates were reported as sensitive to ampicillin, gentamicin, rifampin, vancomycin, erythromycin, streptomycin, trimethoprim/sulfamethoxazole, penicillin and chloramphenicol (34). Simultaneous resistance to two and three drugs tested was obsereved in one isolates (7.7%) for each (Table 4). Results obtained by Lotfollahi et al. (10) confirmed that 5 and 1 isolates (55.6%) and (11.1%) were resistant to 2 and 4 antibiotics respectively. In the end, the MAR index analysis reveals that the studios strains had high MAR index value (> 0.2). 0.2, 0.4 and 0.6 MAR index were detected in 4 (30.7%), strains, one (7.7%) strains and one (7.7%) strains respectively (Fig. 2). A MAR index (> 0.20) antibiotics indicate that bacteria originate from an environment where antibiotics are freely available, leading to a high potential for misuse and a ‘high-risk’ source of contamination (23). This study determined the MAR index of >0.20 for isolates (Fig. 2), It confirmsthat there was widespread use of antibiotics and high selective pressure in Al-Diwaniya population. The MAR indices obtained in present study is a possible indication that a proportion 15.4 of the *L. monocytogenes* strains have been exposed to numerous antibiotics.

## CONCLUSION

We used more than one method to prove virulent *L. monocytogenes* as causative of spontaneous abortion in Iraqi women. The high MAR index identified in this investigation makes it necessary for antibiotic susceptibility testing to be conducted prior to antibiotics treatment. This would not only help in the prudent use of antibiotics but also would restrain the dissemination of antibiotics resistant isolates from these bacteria in Iraqi hospitals as well as in the community.
